# Machine Learning‐Driven Prediction of Microplastic Aging Processes and Environmental Risk Assessment Across Multi‐Media Systems

**DOI:** 10.1002/advs.75906

**Published:** 2026-05-28

**Authors:** Yaping Lyu, Xinran Qiu, Xing Li, Tianhuan Yang, Xuetao Guo, Hao Qiu, Peng Zhang

**Affiliations:** ^1^ State Key Laboratory of Advanced Environmental Technology, School of Environment University of Science and Technology of China Anhui China; ^2^ College of Natural Resources and Environment Northwest A&F University Yangling Shaanxi China; ^3^ School of Environmental Science and Engineering Shanghai Jiao Tong University Shanghai China

**Keywords:** aging trajectories reconstruction, federated learning, machine learning, microplastic aging

## Abstract

Machine learning (ML) holds promise for reconstructing microplastic (MP) aging and assessing risks, but current studies rely on small‐scale, accelerated laboratory datasets and single environmental medium models that miss cross‐media transport and environmental interactions in real‐world MP lifecycles. To realize its potential for reconstructing spatiotemporal aging trajectories and toxicological assessment of MPs, this perspective provides a paradigm shift in ML application from fragmented data‐fitting to a holistic, privacy‐preserving, physics‐aware strategy. A novel probabilistic framework reconstructs the environmental history of field‐sampled MPs through mechanistic fingerprinting, using Bayesian inference to reconcile multi‐evidence signals and improve trajectory models for source attribution and risk assessment. Furthermore, we propose the TRACE framework (TRansport, Aging, Corona, Ecotoxicity), which moves beyond the isolated modeling of aging processes and toxicity endpoints. By integrating physics‐informed models with causal discovery, TRACE captures the reciprocal feedback loops between physicochemical evolution and eco‐corona formation, thereby mechanistically linking surface transformations to biological risks. To support this data‐intensive architecture, we advocate for federated learning (FL) to dismantle privacy barriers. This approach facilitates secure, multi‐institutional collaborative modeling without raw data exchange, harmonizing heterogeneous datasets. Ultimately, this cohesive strategy bridges laboratory‐field disparities, moving toward predictive, evidence‐based, and targeted mitigation efforts in global plastic pollution governance.

## Graphical abstract

This perspective proposes a cohesive machine learning strategy to decode microplastic aging. It advocates for Federated Learning to dismantle global data silos and introduces the TRACE framework (TRansport, Aging, Corona, Ecotoxicity). By integrating physics‐informed modeling with causal discovery, this approach bridges the laboratory‐field gap to enable mechanistic, predictive environmental risk assessment.

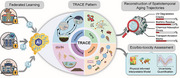

## 1 Introduction

**FIGURE 1:**
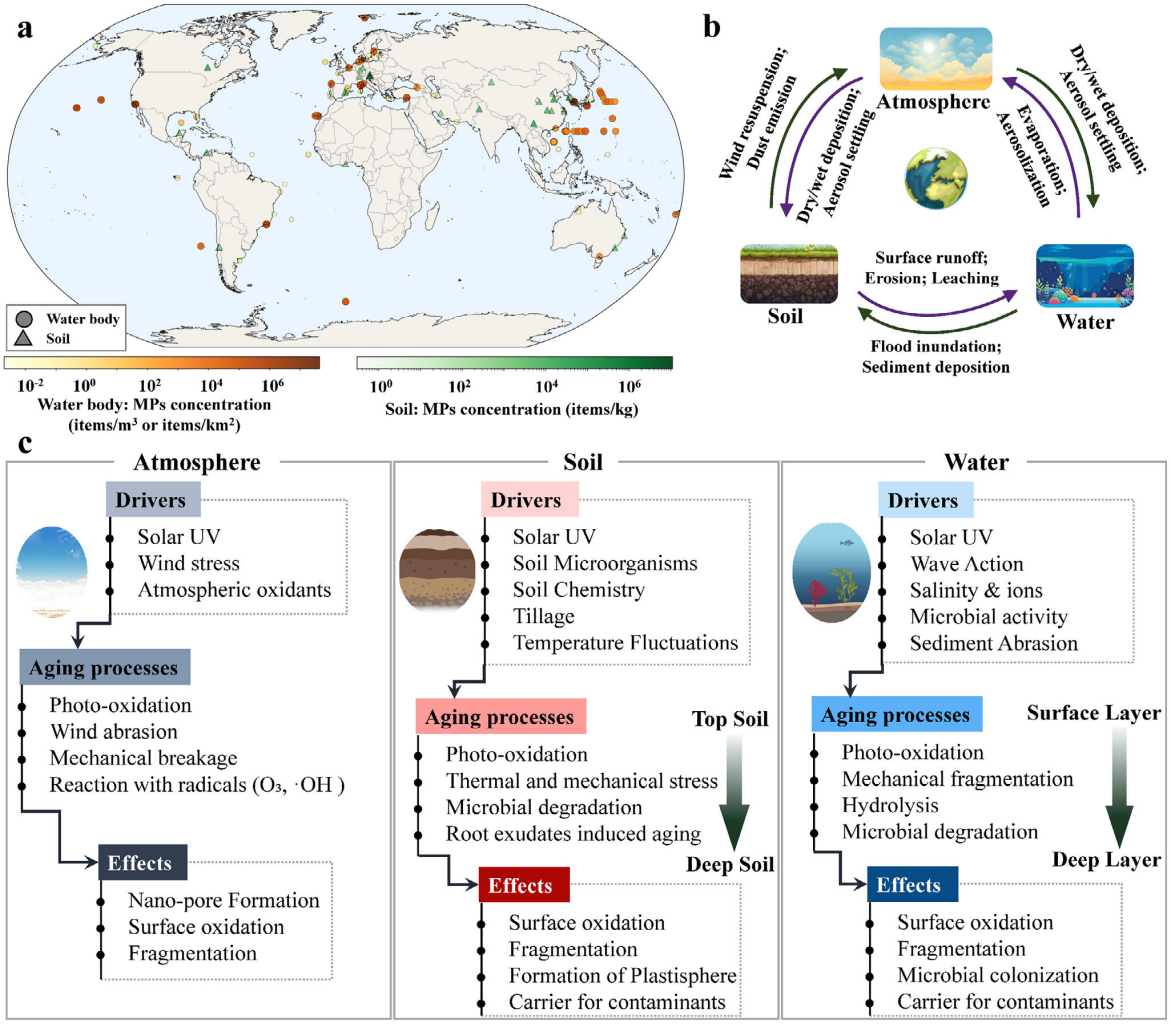
MPs exposure, transport, and aging across the atmosphere, soil, and water. Global distribution of reported MP exposure concentrations in water bodies (circle symbols) and soils (triangle symbols) from the 1990s to present (a). Schematic of pairwise transport pathways and driving mechanisms between atmosphere, soil, and water (b). Comparison of dominant aging drivers and processes in each compartment and their ultimate effects on physicochemical properties of MPs (c).

Microplastics (MPs) contamination has emerged as a widespread issue, with their occurrence documented across aquatic, terrestrial, and atmospheric compartments [1]. While baseline abundances of archived marine samples in the early 1990s were estimated at 0.1–0.2 items/m^3^ [2], current surveys reveal concentrations exceeding 1 × 10^4^ items/km^2^ in impacted coastal systems (Figure 1), with presence extending even to remote regions with minimal human activity, such as Antarctica [3]. Throughout their environmental residency, MPs inevitably undergo aging, which is a process manifesting as surface cracking, introduction of oxygen‐containing functional groups, and reduction in particle size. Aging mechanisms are highly context‐dependent, including photo‐oxidation, mechanical abrasion, thermal degradation, biodegradation, and chemical hydrolysis, each dominating under specific environmental conditions. For instance, in surface water, UV exposure drives photoaging, modulated by fluctuations in temperature, dissolved oxygen, and microbial activity. In deeper aquatic layers, where light is scarce, aging proceeds through microbial degradation and hydrodynamics [4]. Similarly, in surface soils, aging is influenced by sunlight, precipitation, temperature shifts, and soil biota, whereas in subsurface layers, anaerobic microbial activity and chemical hydrolysis prevail [5]. In the atmosphere, UV radiation is the primary driver, synergized by thermal cycling and oxidative pollutants such as ozone, leading to rapid fragmentation and oxidation [6].

Machine learning (ML) algorithms excel at deciphering high‐dimensional, non‐linear relationships from incomplete and heterogeneous datasets. Consequently, they have been increasingly employed to elucidate MP aging processes [7, 8]. For instance, recent studies have utilized ML to reveal the transport mechanisms of aged microplastics in porous media [9] and applied multi‐modal deep learning to trace surface property changes throughout the aging lifecycle [10]. Despite substantial progress in isolating individual mechanisms, current aging research is constrained by several limitations that reduce both ecological relevance and predictive accuracy. First, laboratory simulations employ accelerated aging protocols via intense UV radiation, advanced oxidation processes, or plasma treatments, which fail to capture the synergistic, multi‐factor, and spatially‐temporally variable conditions of natural environments [11]. Real‐world aging involves the simultaneous influence of physical, chemical, and biological processes, modulated by dynamically changing environmental parameters including pH, salinity, temperature, and light intensity [12]. Second, the single‐medium bias ignores the transboundary transport of MPs. Existing ML applications often analyze migration and aging behaviors in isolation, focusing solely on specific compartments such as soil [9] or marine environments [13]. However, as particles transport across aquatic, terrestrial, and atmospheric compartments, they experience distinct aging regimes. These transitions continuously reshape MP surface properties and aging trajectories, while also facilitating entry into biological systems and the food chain, affecting their ecological risks [14]. Finally, these challenges are compounded by practical constraints regarding data availability and standardization, including spatially sparse and temporally uneven field observations, a lack of harmonized metadata standards in sampling and analytical methods, and monitoring time series that are too short to resolve slow, cumulative aging trajectories. These factors create data silos and result in monitoring time series that are insufficient to resolve slow, cumulative aging trajectories. Consequently, these factors result in significant uncertainty in both predicting MP fate and tracing their sources.

The fundamental challenge in MP aging research is insufficient observability and constrained predictability, arising from the synergistic interplay of data sparsity, dispersed data modalities, and process heterogeneity. Addressing this challenge requires a shift in paradigm, moving beyond current ML simulations that rely on single‐medium models and small‐scale laboratory datasets, toward more holistic, multi‐media frameworks. For instance, the persistent gap between laboratory simulations and natural aging can be bridged using domain adaptation and transfer learning techniques. These approaches can integrate controlled experimental data with sparse environmental measurements, learning cross‐domain mappings to predict long‐term aging outcomes under realistic multi‐stressor conditions. Similarly, the lack of dynamic, cross‐media aging studies can be overcome through temporal graph networks or recurrent neural architectures, which reconstruct and forecast aging trajectories as MPs migrate across environmental boundaries. By assimilating data from diverse modalities, including spectroscopy, chromatography, biomolecular assays, and remote sensing, ML models can achieve cross‐modal data fusion, capturing emergent properties that are invisible to single‐technique analysis [10, 15].

ML evolves from a purely descriptive tool into one that can generate hypotheses and guide experimental efforts when it is integrated with mechanistic knowledge and rigorous experimental validation. Well‐designed ML models can produce mechanistically plausible scenarios, help prioritize high‐value field campaigns, and identify compact fingerprints of aging that both improve toxicity inference and support source attribution [16]. Realizing this potential requires harmonized metadata standards, openly shared datasets, and iterative model‐experiment cycles in which ML‐derived hypotheses are rapidly tested and used to refine both models and measurement strategies. In this perspective, we advocate for a cohesive strategy that integrates ML architectures with physicochemical principles. We propose the adoption of Federated Learning (FL) to facilitate secure, multi‐institutional collaborative modeling without the need for raw data exchange. Subsequently, we introduce the TRACE framework (Transport, Aging, Corona, Ecotoxicity), which combines Physics‐Informed Neural Networks (PINNs) with Causal Discovery to mechanistically link physicochemical evolution to toxicological endpoints. Complementing these forward models, we present a probabilistic Bayesian inference framework designed to reconstruct the environmental history of field‐sampled microplastics from non‐unique aging fingerprints. Collectively, these methodological advancements aim to support a transition from descriptive hobservation to predictive, evidence‐based risk assessment.

## 2 Cross‐Modal Data Fusion

Multimodal data integration aims to combine heterogeneous data sources into a unified modeling framework, such as spectroscopic signals, morphological image features, chemical composition analyses, surface physicochemical properties, and ecotoxicological bioassays, while cross‐modal alignment focuses on establishing explicit correspondences or mappings between modalities. Unlike single‐modal analysis, fusion exploits the complementarity between data to reveal intrinsic links between MP origin, aging, transport, accumulation, and ecological risk, enhancing robustness and interpretability in aging‐stage discrimination, source tracing, and risk prediction. For instance, Li et al. employed a multimodal deep learning framework to predict the dominant aging factors of microplastics with 93% accuracy, which is 5–20% higher than the accuracy of a single‐modal model [10]. Similarly, by fusing SEM images with FT‐IR spectra and incorporating the attention mechanism, the accuracy of aging type discrimination is significantly improved [15].

Despite this potential, the application of multimodal models must be fundamentally tailored to the realities of environmental samples. First, multimodal datasets often originate from disparate laboratories, instruments, and experimental protocols, necessitating strictly defined operational standardization to prevent systematic biases during data fusion. When aggregating data across studies, subtle methodological variations manifest as modality‐specific artifacts rather than genuine physicochemical differences [17]. For example, spectral data, such as FT‐IR, Raman, and XPS, may differ by baseline‐correction, smoothing, and normalization strategies or spectral windowing; Numerical data may be reported with different units or hidden preprocessing; and microscopy images, such as SEM and AFM, commonly vary in pixel size, contrast enhancement, and segmentation thresholds. Therefore, data preprocessing must be operationalized distinctly for each data modality to establish uniform preprocessing workflows and harmonized metadata schemas, including spectral, image, numerical, and textual formats. As shown in Table S1, we outline the potential risks of merging heterogeneous multimodal data and propose specific preprocessing mitigation strategies. However, standardizing these workflows at the individual study level is insufficient due to the high time and labor costs involved. It is noteworthy that while data preprocessing reduces uncontrolled variability by representing it in the model as uncertainty or confidence, it does not eliminate modality‐specific biases. Therefore, the persistent lack of large‐scale, well‐annotated multimodal databases is a core challenge. Emerging database initiatives must integrate concrete preprocessing standards to establish a baseline, while simultaneously accepting that models must be mathematically robust to the remaining, irreducible instrumental heterogeneity. Second, label and semantic misalignment leading to shortcut learning and spurious correlations, which occur when a model achieves high nominal accuracy by latching onto superficial, non‐causal statistical patterns rather than the true underlying physical mechanisms. In scenarios with limited data or low‐noise dominant modalities, simply concatenating features from different modalities and training a classifier leads the model to over‐rely on the easiest‐to‐learn modality. For example, a classifier trained on a mixed dataset may learn to rely almost exclusively on a low‐noise spectral channel, such as strong FT‐IR peaks, while ignoring weaker but mechanistically informative image or metadata features. The resulting model attains high nominal accuracy yet fails to capture causal aging mechanisms [18]. To make these risks concrete, we align semantically equivalent variables across modalities, such as oxidation degree, surface topography, particle size, contaminant loading, and bioavailability, rather than raw sensor outputs.  In Table S2, we provide examples of such semantically aligned variables in the context of MP aging. For instance, a robust model should explicitly align the latent representation of chemical oxidation, such as the carbonyl index at 1715 cm^−1^ extracted from FT‐IR, with the latent representation of physical embrittlement, such as surface crack density or fractal dimension extracted from SEM images. Without explicit alignment and constraints, models risk learning non‐interpretable statistical associations rather than testable mechanistic features. Third, the domain shift from laboratory to field conditions is uncontrollable. Controlled accelerated‐aging experiments differ fundamentally from field samples in terms of exposure regimes, co‐occurring contaminant spectra, and histories of mechanical abrasion [12]. In the natural environment, MP aging is driven by a combination of physical, chemical, and biological mechanisms [19, 20] and proceeds in a much slower and more complex manner than in laboratory conditions [21]. Environmental factors such as pH, salinity, temperature, and UV intensity dynamically influence this aging process [22, 23]. Models trained exclusively on laboratory data often perform poorly on environmental samples. Yet, systematic work on domain adaptation, calibration transfer, or embedding physical priors to enhance extrapolation capability remains scarce [24]. Fourth, intrinsic data properties in spectroscopic and microscopic modalities dictate that low signal‐to‐noise ratios are often unavoidable. Noise, baseline drift, spectral overlap, scattering, and occlusion artifacts in micrographs are often underestimated. While ML–based denoising or artifact removal has been proposed to enhance spectral usability, ML frameworks must be inherently designed to handle noisy, low‐resolution inputs without cascading error [25].

To address these intrinsic constraints, progress must be made on both data and algorithmic fronts. Beyond promoting data standardization and sharing, model design should evolve from feature concatenation to semantic alignment and physical constraints. Techniques such as cross‐modal alignment [26], contrastive learning [27], and cross‐modal knowledge distillation under self‐/weakly supervised settings can establish semantic correspondences between modalities. Simultaneously, known physicochemical principles, such as cumulative dose–response relationships in photodegradation, Arrhenius‐type temperature dependence of rate constants, or qualitative traits of adsorption isotherms, should be embedded as engineered features or soft constraints in the model loss function. This ensures that, even with limited data, models remain grounded in mechanistic causal pathways. In addition, domain adaptation and uncertainty quantification should be treated as standard components of cross‐modal frameworks. During training, adversarial domain adaptation [28], distribution alignment [29], or feature normalization–based calibration transfer can be applied. Output probabilistic confidence intervals at inference using deep ensembles and MC‐dropout are suggested to clearly delineate model applicability boundaries and high‐uncertainty regions requiring targeted sampling. To address the challenges of label noise and sparsely labeled data in real‐world scenarios, we propose integrating recognition, aging stage classification, adsorbate spectrum prediction, and toxicity endpoint estimation into a joint multi‐task learning framework. This framework facilitates shared representation learning across tasks. We further leverage unlabeled or weakly labeled field data to enhance the learning signal through contrastive learning or pseudo‐labeling techniques. Coupled with an active learning strategy, we prioritize labeling samples based on model uncertainty estimates. This approach strategically targets samples most informative for cross‐modal alignment and domain adaptation, thereby maximizing data utility and model performance under constrained experimental budgets.

## 3 Reconstruction of Spatiotemporal Aging Trajectories

MPs aging is a continuous and dynamic process characterized by significant spatiotemporal heterogeneity. The reconstruction of spatiotemporal trajectories can reveal critical transition points during migration across different environments. The experiences of an MP particle determine its physicochemical characteristics, including its size distribution, surface functional groups, adsorbed contaminant profile, and bioavailability [30]. These characteristics directly influence exposure‐response relationships at both individual and population levels. Consequently, risk assessments based on trajectory reconstruction allow researchers to infer potential antecedent drivers, such as cumulative UV dose, wet–dry cycles, and microbial exposure duration, based on the current state characterization of a sample [10]. This retrospective analysis provides a quantitative basis for identifying key control points, such as blocking specific migration pathways.

For management and monitoring, trajectory models have the potential to prioritize geographical regions or temporal windows prone to forming high‐risk convergence zones, thereby optimizing sampling design, resource allocation, and intervention strategies. Existing spatiotemporal research on MPs predominantly focuses on concentration variations or transport pathway simulations, while dynamic modeling of physicochemical property evolution over time and across environments remains nascent or limited to localized applications [31]. Mathematical models have been employed to track MP transport and fate in the environment, often coupled with processes like fragmentation or sedimentation [10, 32]. These studies provide the foundational physical framework for trajectory reconstruction. Reliable spatiotemporal aging trajectory reconstruction requires building upon this foundation by incorporating systematic aging kinetic parameters and integrating sufficient field observations for calibration.

Aging encompasses chemical‐physical degradation driven by irradiation, temperature, and moisture fluctuations, and mechanical energy, alongside biochemical transformations mediated by microorganisms and interfacial processes involving contaminant adsorption–desorption. These mechanisms exhibit significant spatial and temporal variability influenced by climate, geomorphology, land use, and anthropogenic disposal history [33]. Consequently, trajectory reconstruction requires not only high‐resolution physicochemical characterization of samples at discrete time points but also recovery or estimation of the environmental exposure history of the sample. This history includes parameters like cumulative UV dose, thermal‐hydraulic cycles, salinity and pH conditions, flow velocity and shear stress, microbial activity indicators, and co‐contaminant profiles. Achieving this necessitates the integrated and unified interpretation of discrete observational data and underlying mechanisms. ML offers the capacity to synthesize existing discrete observations and simulation data with spatial information, including sampling location coordinates, topographic or hydrological attributes, and nearby potential source inventories [34]. Furthermore, it integrates temporal information such as sampling dates, known anthropogenic activities, or extreme meteorological events to enable the interpolation and prediction of aging processes. This involves deep characterization of spectroscopic and microscopic imaging data to quantify interfacial properties and particle size distributions, and incorporating environmental sequence information represented as time intervals or cumulative doses into models, constituting a typical multi‐modal, variable‐length sequence inference problem. Reconstructing historical trajectories from a final observed state introduces severe identifiability challenges. Distinct environmental exposure histories, such as a short burst of intense UV radiation vs. prolonged exposure to moderate abrasion and chemical oxidation can result in nearly identical terminal physicochemical states. Acknowledging this fundamental limitation, trajectory reconstruction cannot rely on deterministic point estimates, as they would falsely project certainty where multiple valid historical paths exist. Instead, the framework must be formalized as a Bayesian inference problem. By outputting a probabilistic distribution of plausible spatiotemporal pathways rather than a single route, the model explicitly quantifies the identifiability gap. This probabilistic treatment highlights specific trajectory segments where overlapping mechanisms prevent unique historical attribution, thereby guiding researchers to select complementary fingerprints to further constrain the solution space.

**FIGURE 2:**
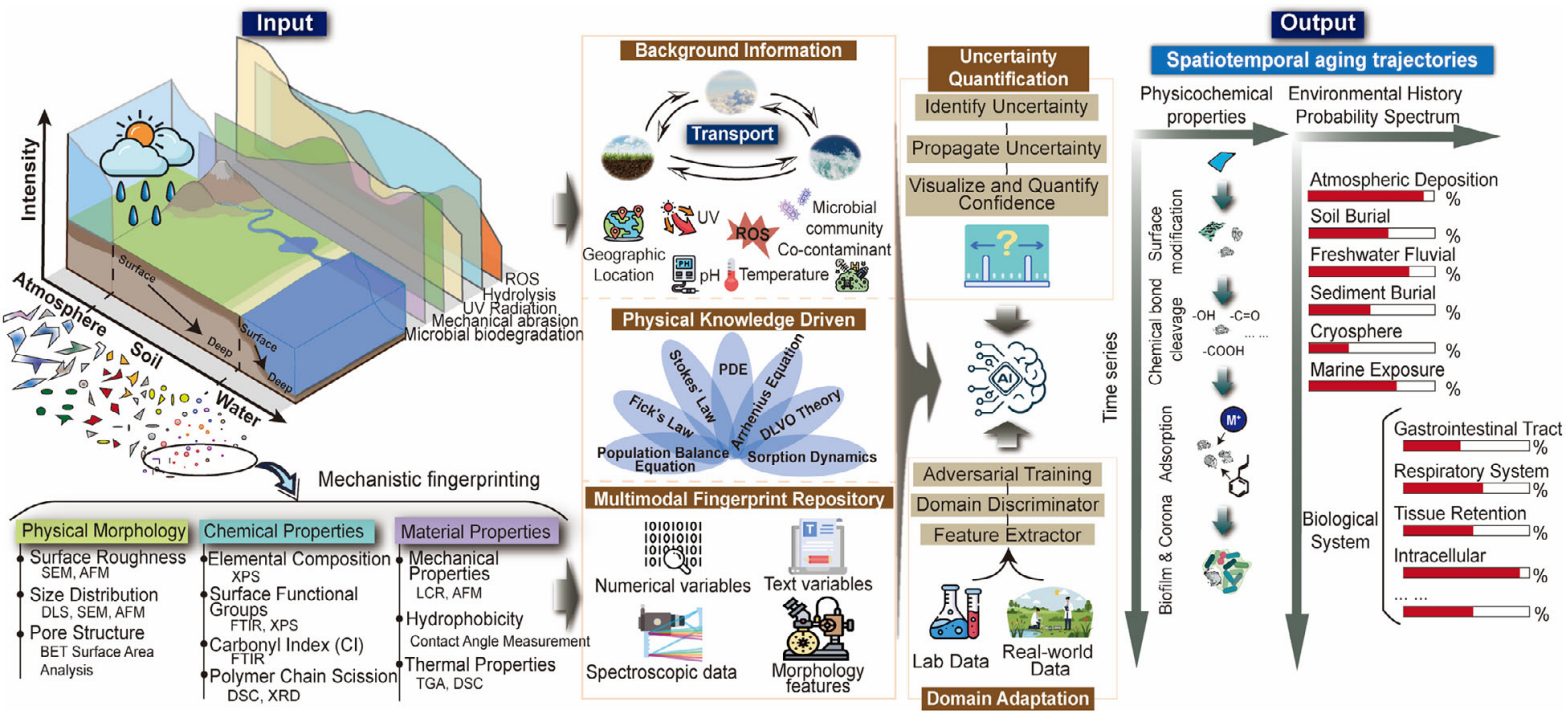
A multi‐modal, physics‐informed learning framework for spatiotemporal reconstruction of MP aging trajectories. This framework integrates aging fingerprints, discrete environmental measurements, and spatiotemporal context in a unified machine learning architecture. The model leverages independent representation learners for each modality, fuses them with temporal encodings, and embeds physical priors to constrain the solution space. The final output provides a probabilistic reconstruction of the aging trajectory, incorporating uncertainty quantification and domain adaptation to enable robust prediction and source attribution across varying environmental conditions.

We propose addressing this issue from five aspects, including data strategy, model architecture, incorporating physical knowledge, addressing domain shift, and uncertainty quantification (Figure 2). Regarding data strategy, we advocate supplementing the controlled laboratory accelerated and gradient aging experiments with field‐collected and long‐term in situ observation data. Field data serve crucial roles for out‐of‐domain testing and model calibration, serving as sources for semi‐supervised or weakly supervised learning. In terms of model architecture, we propose a robust strategy involving initial independent representation learning for each modality. For example, 1D‐CNNs and Transformers are suitable for spectral data, 2D‐CNNs for microscopic images, and tree‐based models or fully connected networks for numerical features. Subsequently, these intermediate representations are fused using sequence models, such as Long Short‐Term Memory (LSTM) networks or Temporal Convolutional Networks (TCN), which incorporate time and phase encodings. Because microplastics undergo dynamic transitions in dominant weathering processes as they cross environmental boundaries, these sequence models are critical. Rather than relying on static, isolated indicators, they capture the temporal evolution of aging by continuously evaluating shifts in the combinatorial profiles of multimodal features, thereby naturally reflecting the complex interplay and transition of underlying physicochemical processes over time. The output is a probabilistic reconstruction of the trajectory, providing the most probable sequence of environmental segments and parameter estimates for each segment. Crucially, physical priors should be embedded within the model to ensure these sequences obey established degradation kinetics. As detailed in Table S3, representative physical laws, such as Arrhenius temperature dependencies for chemical oxidation, Population Balance Equations (PBE) for mechanical fragmentation, or Monod kinetics for bio‐corona growth, can be formulated as penalty terms. This forces the model to output degradation rates and state transitions that strictly obey thermodynamic and kinetic boundaries, mitigating the fragility of purely data‐driven models during extrapolation. To handle inherent domain differences between controlled experiments and field samples, domain adaptation or transfer learning strategies should be employed. These techniques transfer mappings learned under controlled conditions to field scenarios, using limited high‐quality field‐annotated samples for fine‐tuning or correction. Finally, given the critical importance of uncertainty information in trajectory reconstruction, model outputs should adopt probabilistic representations, such as Bayesian networks, deep ensembles, and Monte Carlo dropout. By adopting probabilistic representations, the model inherently absorbs these analytical errors, translating input noise into appropriately widened confidence intervals for the predicted pathways rather than causing catastrophic predictive failures. This probabilistic approach is particularly essential because reconstructing historical trajectories from a terminal observation often encounters equifinality, where divergent environmental exposure histories produce analogous final physicochemical properties. By outputting a distribution of plausible pathways rather than a single deterministic route, the model explicitly accounts for this non‐uniqueness. These probabilistic outputs, coupled with interpretability tools such as SHAP values [35], attention visualization [36], and integrated gradients [37], allow researchers to map key drivers inferred by the model back to measurable physicochemical quantities, thereby generating verifiable mechanistic hypotheses. By capturing trends in aging indicators through temporal models or inferring regional aging potential from environmental data, MP aging pathways can be reconstructed at macro scales. Such reconstructions not only facilitate the extrapolation of experimental findings to broader spatiotemporal contexts but also elucidate the impacts of long‐term cumulative effects and transboundary migration on MP aging.

## 4 Probabilistic Tracing From Fingerprints

Systematic characterization and calibration reveal that MP aging leaves a rich set of mechanistically interpretable fingerprints, which can be leveraged to trace a particle's prior environmental exposures [38, 39]. This evidence‐based tracing approach complements broader trajectory models by providing mechanistic constraints, thereby rendering spatiotemporal reconstructions more robust and actionable. However, given that different aging mechanisms often leave overlapping fingerprints, the interpretation of evidence must be probabilistic rather than deterministic.

Distinct environmental processes impart specific and detectable signatures. Photo‐oxidation and photochemical aging driven by solar irradiation introduce oxygenated functionalities, increase near‐surface oxygen‐to‐carbon ratios, and generate low‐molecular‐weight oxidation products that may leach into surrounding media. Such signatures are quantifiable via changes in Fourier‐transform infrared (FTIR) and Raman spectral peaks, specifically the growth of carbonyl bands, as well as through elevated O/C ratios in x‐ray photoelectron spectroscopy (XPS) [40]. Abrasion and mechanical fatigue generate characteristic morphological signatures, such as surface micro‐cracks, increased surface roughness and specific surface area, and altered particle size distributions, which can be observable by SEM, AFM, and particle sizing instruments [40]. Bio‐mediated transformations resulting from microbial colonization and enzymatic degradation alter surface hydrophobicity, introduce biogenic organic signatures, such as extracellular polymeric substance residues, and can selectively transform additives or small polymer fragments. Biofilm presence and composition are detectable via microscopy, targeted biomolecular assays, and solvent extract profiling [41, 42]. In soils and sediments, chemical oxidation mediated by reactive oxygen species or catalysis at mineral surfaces becomes relatively important where UV is attenuated, and can leave mineral residue fingerprints or specific oxidation products detectable by EDS, XPS, and mass spectrometry [11].

Critically, these fingerprints are often non‐unique, as different mechanisms can produce overlapping features. For example, oxidation features can arise solely from UV exposure or following mechanical abrasion that exposes fresh polymer. Therefore, tracing must treat evidence probabilistically rather than deterministically. Each measured indicator should be converted to a probabilistic evidence term. This can be formalized using Bayes’ theorem, where the posterior probability *P(E∣H)* of a candidate exposure history *H* given the observed evidence *E*is proportional to the product of the likelihood *P(E∣H)* and the prior probability *P(H)* (Equation (1)) [43, 44]. Here, E denotes the observed fingerprint vector, including spectroscopic, morphological, chemical, and compositional descriptors, whereas H represents a candidate exposure‐history hypothesis, such as marine surface exposure, soil burial, or atmospheric transport. The likelihood P(E∣H) should be interpreted as a calibrated conditional distribution, and it quantifies the probability of observing the measured fingerprints under a given exposure scenario, while the prior P(H) incorporates coarse contextual constraints such as sampling location and hydrological data. In practice, this likelihood can be estimated from replicated controlled aging experiments under known conditions and then calibrated using field‐anchored samples with constrained or known histories. This framework naturally handles complex and conflicting evidence. For example, a combination of an elevated carbonyl index and marine salt residue strongly supports a history of marine surface exposure, whereas high crack density with low oxidation indicates a mechanically dominated history. The reliability of this approach depends on accurately calibrated likelihood functions, derived from controlled laboratory aging experiments and validated against field‐anchored marker samples with known histories. This probabilistic formulation allows the framework to explicitly represent uncertainty, resolve non‐unique historical reconstructions, and support evidence‐based inference of MPs’ environmental pathways.

(1) P(H∣E)∝P(E∣H)P(H)

## 5 Integrating Corona Evolution Into the Spatiotemporal Tracing

Eco‐corona and bio‐corona represent the dynamic layers of natural organic matter, ions, pollutants, microbial extracellular polymeric substances (EPS), and biomolecules that coat micro‐ and nanoplastic surfaces [45]. These coronas, while not traditional aging fingerprints, are inextricably linked to aging processes that critically determine the environmental fate and risks of MPs. Crucially, coronas are not passive coatings, and they actively modulate MP aging trajectories through complex, context‐dependent feedback loops. In a predictive framework, therefore, corona composition should not be treated as a static annotation but as a time‐varying state variable that jointly evolves with polymer aging. This perspective is important because corona formation can both accelerate and suppress aging kinetics depending on the environmental context. Adsorbed organic matter or microbial biofilms can accelerate surface oxidation via photosensitization. Conversely, they can also inhibit photodegradation by shading the polymer surface or scavenging radicals [46]. Similarly, biofilm‐derived enzymes and metabolites can catalyze polymer chain scission, yet a thick corona may also limit oxygen and light penetration, thereby slowing oxidative breakdown. The corona formation also significantly affects the environmental transport behavior of MPs by altering hydrophilicity/hydrophobicity and changing physical straining in environments [47]. This dynamic interplay collectively determines the long‐term evolution of particle size, surface chemistry, and mechanical integrity, further controlling bioavailability at multiple stages. These opposing effects suggest that aging models should allow corona descriptors, such as EPS thickness, protein‐to‐carbohydrate ratio, zeta potential, and NOM loading, to enter the kinetic module as dynamic covariates that modulate effective rate constants rather than as post hoc explanatory variables. In this way, the model can represent the corona as a latent interfacial state that reshapes the trajectory of surface oxidation, fragmentation, and transport.

The same coupling operates in the reverse direction. Aging reshapes corona formation and function. Aging‐induced increases in surface polarity and specific surface area tend to enhance sorption of organic contaminants and metal cations, thereby changing the composition and stability of subsequent corona layers. Experimental aging studies report multi‐fold increases in adsorption affinity for model contaminants, such as methylene blue and polycyclic aromatic hydrocarbons (PAHs), on weathered polyethylene and polypropylene compared to pristine counterparts [48]. This enhanced loading converts aged particles into more effective vectors for co‐contaminants, increasing the mass of potentially bioavailable toxicants delivered to organisms upon ingestion or inhalation. This process not only increases contaminant loading but also changes the features that should be learned by predictive models, because the corona becomes part of the particle's evolving state rather than a separate external layer. Accordingly, a useful modeling strategy is to jointly encode aging fingerprints and corona composition into a shared latent representation, with oxidation‐related variables, morphological descriptors, and corona chemistry aligned across modalities. Such a design allows the model to learn the co‐evolution of surface chemistry and interfacial loading, while reducing shortcut learning that would arise if corona signals were treated independently from the aging state. Furthermore, changes in surface topography alter biological recognition. It has been proven that the protein corona is markedly different between environmentally realistic fragmentation‐derived nanoplastics and model spheroidal nanoparticles, likely leading to different internalization and downstream signaling pathways [49]. From a modeling perspective, this means that the corona state should be represented as a mechanism‐sensitive feature influencing both transport and bioavailability, rather than simply as a descriptive endpoint. For example, corona descriptors can be used to parameterize a latent interfacial affinity or bioavailability state that mediates organism–particle interaction and contaminant delivery. This same latent state can then be coupled to toxicity prediction or trophic transfer models, enabling a unified framework that connects aging, corona assembly, and ecological effects within a single predictive structure.

This feedback loop extends to trophic transfer and ecological cycling. Following ingestion by organisms, MPs may either be eliminated via excretory pathways or translocated to other organisms via the food chain. For example, when lettuce contaminated with nanoplastics is ingested by snails, a fraction of the NPs is retained and accumulated in the snail's tissues, while the remainder is excreted in feces and re‐enters the soil matrix [50]. The bio‐corona formed on these retained particles may further modulate their subsequent environmental behavior and fate, both within the organism and upon re‐entry into ecological systems. From a risk‐assessment perspective, laboratory studies that use pristine MPs in simple media risk underestimating or mischaracterizing real‐world corona formation, aging dynamics, and the consequent bioavailability patterns. It is highly suggested that models for tracing MP fate and risk must account for the interaction between aging processes and corona dynamics.

Integrating corona evolution into spatiotemporal tracing presents distinct challenges. The primary difficulty lies in the dynamic and reversible nature of coronas [51]. As MPs transit through environments and organisms,  the initial corona can be displaced by molecules from subsequent environments. Aging further alters the MP surface chemistry, which in turn decides the composition and conformation of the adsorbed biomolecular layer, exacerbating identification uncertainty. In practice, this means that a single molecular memory signal is unlikely to be stable across time and space; instead, the data are better viewed as a sequence of corona states evolving under changing exposure histories. A model built for such data should therefore preserve this temporal structure, for example, by using a state‐space or switching framework in which corona composition and aging state are jointly inferred from paired observations. This is also where harmonized metadata and co‐collected multimodal measurements become essential. If aging descriptors, corona composition, and exposure history are recorded on the same particles or paired aliquots, then the model can learn to distinguish true mechanistic transitions from sampling noise or cross‐site variation. Under this design, data standardization is not an end in itself, but a prerequisite for learning coupled aging–corona dynamics with calibrated uncertainty.

Controlled laboratory experiments, while essential, often fail to reproduce the multi‐scale process evolving synchronously in time and space. It also creates domain gaps when extrapolating results to field systems where drivers are high‐dimensional, non‐stationary, and nonlinear in their interactions. Recent work demonstrates robust ML performance on concrete subproblems relevant to aging and corona formation [15]. Here, we suggest modeling aging and corona processes within a unified, cross‐scale framework designed specifically to enhance the mechanistic robustness of predictive aging models. First, aging and corona measurements should be systematically co‐collected on the same particles or paired aliquots, supported by harmonized metadata that document exposure history, analytical parameters, and environmental context. Establishing such paired and temporally aligned observations ensures that transformations in polymer chemistry, surface functionality, and corona composition can be interpreted within consistent spatiotemporal coordinates. Second, aging metrics and corona compositional features must be standardized, quality‐controlled, and converted into physically interpretable descriptors. While cumulative exposure metrics are derived to capture process intensities more faithfully than discrete snapshot conditions. Third, aging‐ and corona‐related variables should be integrated into a joint structured dataset that retains both raw multimodal information and domain‐informed engineered features, enabling subsequent models to exploit complementary mechanistic signals and cross‐modal correlations. Operationally, this enables subsequent ML models to exploit complementary mechanistic signals by treating corona metrics as coupled state variables that continuously update the degradation rate laws. Finally, by embedding these harmonized and co‐registered data streams into a single modeling matrix, ML approaches can more reliably disentangle the reciprocal influences between aging dynamics and corona assembly, mitigate laboratory‐to‐field domain gaps, and provide robust, generalizable predictions of environmentally relevant transformation pathways.

## 6 Holistic Toxicity Assessment of Aged MPs

Aging fundamentally reshapes the toxicological profile of MPs. It alters not only the physical morphology and chemical composition of MPs but also reshapes their bioavailability, contaminant‐carrying capacity, and interactions with biological interfaces. Fresh MPs are typically characterized by larger fragments or fibers, relatively low surface polarity, and the presence of inherent additives. Aging induces multiple transformations in materials, including surface oxidation with an increase in polar functional groups such as carbonyl and carboxyl, micro‐crack formation with heightened surface roughness, a particle size distribution shift toward finer fragments, elevated specific surface area, altered surface charge and hydrophilic character, and the leaching or chemical transformation of additives and low‐molecular‐weight components. These changes collectively modify the absorption, accumulation, and toxic pathways within exposed organisms. Naturally aged MPs are more susceptible to mechanical fragmentation into smaller particles [52], which facilitates their uptake into plant tissues [53], enables penetration of cellular membranes [54], and induces elevated levels of reactive oxygen species (ROS), thereby causing more severe cytotoxic effects [55]. It is indicated that aging significantly aggravated MPs biotoxicity in algae, benthic invertebrates, zooplankton, and fish [14]. Negative effects are reflected in growth and development, behavioral, sensory, and neuromuscular functions, metabolism, alimentary and excretory systems, oxidative damage, and plastic ingestion [56].

**FIGURE 3:**
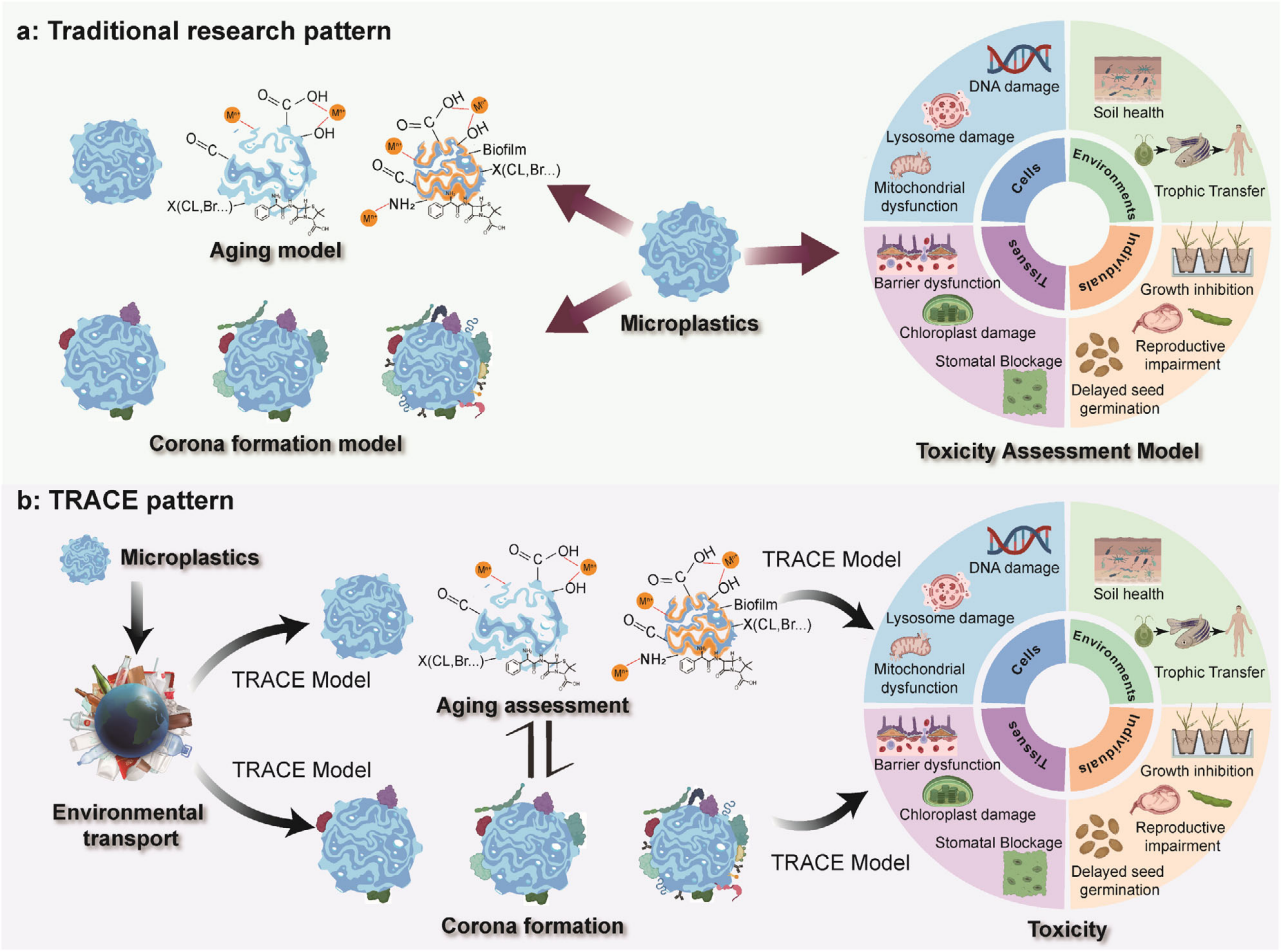
Traditional research pattern (a) and TRACE pattern (b) in toxicity assessment of Aged MPs. TRACE pattern is a unified framework to bridge the fragmentation in current MP research. Unlike a conventional isolated research pattern, TRACE integrates TRansport, Aging assessment, Corona formation, and Ecotoxicity into a coupled computational pipeline. By capturing the conditional dependencies between physicochemical aging and bio/eco‐corona acquisition, this pattern provides a holistic prediction of microplastic toxicity, elucidating how environmental transformations dynamically alter the biological fate of MPs.

Currently, most toxicology studies typically report organismal and cellular endpoints, such as LC/EC values, growth inhibition, ROS, membrane damage, and biomarker changes, from exposures that often use commercial or simply‐prepared MPs (Figure 3a). Research on the toxicity of aged MPs remains limited and fragmented, with a critical lack of integrative toxicological frameworks that simultaneously account for environmental variables, MP properties, and species‐specific characteristics. Aging analyses catalogue detailed physicochemical descriptors but rarely link them quantitatively to biological endpoints. Similarly, corona metrics are rarely incorporated into ecotoxicity models along with aging descriptors, which strongly affect MP stability and bioavailability. Moreover, environmental behavior and biological effects of MPs involve multiple scales from the molecular to the ecosystem, creating challenges for cross‐scale integration in toxicity assessment. Specifically, the toxic effects include oxidative stress and inflammatory responses at the cellular level, tissue damage at the organ level, and impacts on growth, reproduction, and behavior at the individual level. This multi‐scale complexity, combined with fragmented data and methodologically narrow study designs, highlights the urgent need for more unified and predictive approaches to fully elucidate the ecotoxicological significance of MP aging.

ML offers a promising approach to construct intelligent mapping networks linking aging characteristics, corona formation metrics, environmental parameters, and toxicity responses. A primary issue is the difficulty in data sharing across institutions due to privacy and ownership concerns, which hinders the aggregation of datasets. Generating the high‐fidelity multimodal datasets required for comprehensive MP assessment, particularly long‐term ecotoxicological bioassays paired with continuous high‐resolution microscopic and spectroscopic tracking, is exceptionally resource‐intensive. Consequently, research institutions are often hesitant to share raw, proprietary datasets due to intellectual property considerations, the competitive landscape of academic publishing, and the massive storage requirements of raw multimodal files. This inherently creates data silos, severely restricting the sample sizes and environmental heterogeneity available to train robust, globally applicable models. To overcome this limitation, federated learning (FL) provides a privacy‐preserving strategy that enables collaborative model training across institutions without requiring the exchange of raw MP datasets [57]. By allowing models to be trained locally and only sharing aggregated updates, FL effectively addresses data silos while preserving data ownership and confidentiality. Specifically, secure aggregation ensures that individual updates remain unidentifiable during the aggregation process [58], while differential privacy introduces calibrated noise to prevent the inference of specific samples from the final model [59]. Furthermore, homomorphic encryption allows computation on encrypted updates [60], and TEEs offer hardware‐based secure enclaves for processing [61]. By decentralizing the training process, federated learning incentivizes broader institutional participation, allowing the global TRACE framework to learn from diverse, high‐value datasets while strictly preserving the ownership and confidentiality of the localized raw data. Nevertheless, practical implementation of FL may face challenges such as system heterogeneity across institutions, communication overhead, and inconsistencies in data quality and measurement protocols, which require careful coordination and standardization. Additionally, algorithms must be explicitly designed to handle non‐independent and identically distributed data, as individual laboratories often possess highly skewed datasets biased toward specific local environments, polymer types, or target organisms.

**FIGURE 4:**
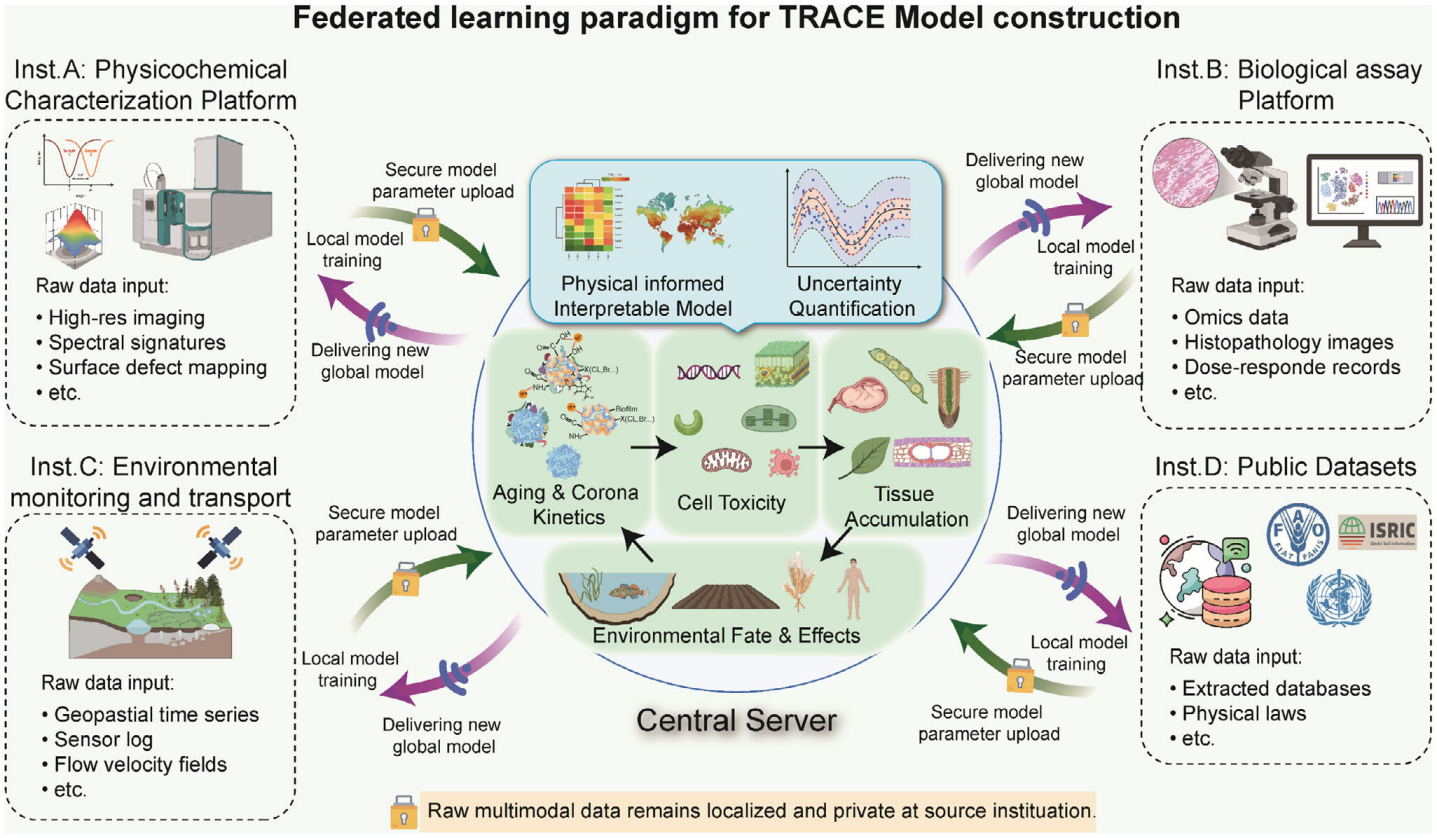
Schematic overview of federated learning paradigm for TRACE model construction, which integrates data from four distributed sources (Institutions A, B, C, D, etc.) using local training modules equipped with Physics‐Informed Neural Networks. Encrypted gradients are sent to the central TRACE server to update the global causal model for multiscale causal discovery in MPs toxicology, while raw multimodal data remains private.

To address the current fragmented understanding of MP aging, bio/eco‐corona formation, and ecotoxicological effects, we propose an integrative causal network that links transport, aging assessment, corona formation, and ecotoxicity (TRACE framework, Figure 3b). The core is the construction of a model that intrinsically couples environmental spatiotemporal dynamics, particle physicochemical evolution, and multiscale biological responses of MPs. Multimodal data fusion techniques are employed to generate a high‐dimensional feature vector that comprehensively represents the aging state and corona formation of MPs (Figure 4). By integrating physics‐informed neural networks with causal discovery algorithms, the model adheres to fundamental physicochemical principles while automatically identifying key causal pathways that drive toxicological endpoints from both measured data and model predictions. It is critical to acknowledge that causal inference is notoriously sensitive to measurement error and analytical noise, which can introduce unobserved confounding and generate spurious causal edges. Therefore, to maintain stability, the implementation of TRACE must incorporate latent variable causal models or robust constraint‐based algorithms that explicitly account for measurement uncertainty. This ensures that the identified toxicological pathways remain stable and mechanistically valid even when analyzing noisy environmental data. Furthermore, interpretability techniques such as counterfactual reasoning enable attribution analysis of model predictions, quantitatively decomposing the relative contributions of environmental processes, interface transformations, and biological response mechanisms to observed toxic outcomes. By closing the loop from physico‐chemical characterization to causal model discovery and interpretable attribution, the framework moves beyond descriptive, indicator‐level studies toward mechanistic, whole‐chain dynamic simulation and quantitative attribution, delivering a practical computational tool for more precise assessment and prediction of MP environmental risk.

## 7 Outlook

MP aging is an inherently multiscale problem where physical degradation, chemical transformation, and biological interaction evolve synchronously. While ML has demonstrated strong capability in fitting correlations, the field stands at a critical transition point. Future research must address three strategic imperatives. First, redefining data collaboration through privacy‐preserving paradigms. The current reliance on fragmented, single‐lab datasets limits model generalizability. The immediate priority is not merely to collect more data, but to establish FL ecosystems. By standardizing annotation schemas and metadata protocols, researchers can train global, robust models across institutions without compromising data privacy or ownership. Second, evolving from “black‐box” prediction to physics‐informed causal discovery. Naive concatenation of multimodal features is insufficient for unraveling complex mechanisms. Future architectures must explicitly embed physicochemical constraints directly into the loss functions of neural networks. This integration is the most reliable route to distinguish between spurious statistical correlations and actual drivers of toxicity, thereby generating interpretable, mechanistically grounded hypotheses. Third, closing the loop on dynamic risk assessment and real‐world validation. The static approach to toxicity must be replaced by dynamic trajectory modeling. Models should focus on the reciprocal feedback between aging and corona evolution, quantifying how surface transformations dynamically modulate bioavailability and contaminant transport over time. This includes refining the probabilistic Bayesian frameworks to trace sources from non‐unique fingerprints, providing evidence needed for pollution control. Crucially, Validation of the proposed framework should extend beyond laboratory benchmarks to external field samples spanning multiple sites, seasons, and environmental compartments. Performance evaluation should therefore combine predictive accuracy, uncertainty calibration, and cross‐domain robustness under realistic environmental variability. In summary, ML stands to revolutionize our understanding of MP aging, but its success hinges on collaborative, interdisciplinary efforts that integrate domain knowledge, prioritize data quality, and emphasize ecological relevance. Advancing these directions will move microplastic research from descriptive analytics to predictive, actionable science that supports evidence‐based environmental management and risk mitigation.

## Author Contributions

**Xing Li**: investigation, validation, writing – review and editing. **Tianhuan Yang**: investigation. **Hao Qiu**: writing – review and editing, validation. **Xuetao Guo**: writing – review and editing, visualization. **Xinran Qiu**: investigation, validation. **Yaping Lyu**: investigation, writing – original draft, visualization, writing – review and editing, methodology, validation. **Peng Zhang**: funding acquisition, investigation, validation, methodology, visualization, writing – review and editing, supervision.

## Conflicts of Interest

The authors declare no conflicts of interest.

## Supporting information




**Supporting File**: advs75906‐sup‐0001‐SuppMat.docx.

## Data Availability

The data that supports the findings of this study are available in the supplementary material of this article.
