# Supplementary material for: Machine Learning‐Driven Prediction of Microplastic Aging Processes and Environmental Risk Assessment Across Multi‐Media Systems
Source: Adv Sci (Weinh). 2026 May 28;13(36):e75906. doi: 10.1002/advs.75906 (PMC13317599; doi:10.1002/advs.75906)
Supplement: Supplementary file 1 — Supporting File: advs75906‐sup‐0001‐SuppMat.docx. [file ADVS-13-e75906-s001.docx]

**Machine Learning-Driven Prediction of Microplastic Aging Processes and Environmental Risk Assessment Across Multi-Media Systems**

*Yaping Lyu^1^, Xinran Qiu^2^, Xing Li^3^, Tianhuan Yang^3^, Xuetao Guo^2*^, Hao Qiu^3*^, Peng Zhang^1*^*

*^1^* *State Key Laboratory of Advanced Environmental Technology, Department of Environmental Science and Engineering, University of Science and Technology of China, Anhui, 230026, China*

*^2^ College of Natural Resources and Environment, Northwest A&F University, Yangling, Shaanxi, 712100, China*

*^3^ School of Environmental Science and Engineering, Shanghai Jiao Tong University, Shanghai, 200240, China*

**Corresponding author. E-mail address:* [*guoxuetao2005@nwafu.edu.cn*](mailto:guoxuetao2005@nwafu.edu.cn) *(X. G);* [*haoqiu@sjtu.edu.cn*](mailto:haoqiu@sjtu.edu.cn) *(H. Q); zhangpeng1987@ustc.edu.cn (P. Z);*

**Table S1 Proposed operational data preprocess and harmonized metadata schema across data modalities for MP aging modeling.**

| **Data Type** | **Key problems** | **Operational preprocessing** |
| --- | --- | --- |
| Numerical data | 1. Unit and aggregation mismatch  2. Different precision and missingness patterns.  3. Hidden preprocessing leading to incompatible columns.  4. Collinearity and differing sampling frames. | 1. Enforce unit standardization at ingest and record conversion in metadata; keep raw values plus standardized column.  2. Schema and controlled fields. Require measurement method, aggregation rule, n rep, etc. fields.  3. Imputation and missingness handling. MICE, KNN, or model-based imputation with uncertainty estimates; flag imputed values.  4. Feature harmonization and selection. Compute comparable summary descriptors, remove collinearity by PCA or domain-knowledge feature pruning.  5. Hierarchical and Bayesian models for pooling across sites while modeling site effects explicitly. |
| Textual Data | 1. Inconsistent vocabulary, synonyms and typos.  2. Missing required metadata.  3. No provenance or pipeline versioning, rendering the results irreproducible. | 1. Controlled vocabulary and minimal schema. Define required fields and use JSON Schema for validation.  2. Text normalization pipeline. For example, lowercase, dictionary mapping of synonyms, and unit normalization.  3. NLP extraction for historical text. Extract structured fields from free-form text by combining regular expressions with a lightweight rule-based parser.  4. Label quality control. Consensus labeling, calculate inter-rater kappa, or use noise-robust loss and co-teaching in model training. |
| Spectral data | 1. Heterogeneous preprocessing. Different baseline correction, smoothing, windowing, normalization led to peak shifts, amplitude and scaling differences.  2. Differing wavelength bins.  3. Feature-level semantic mismatch.  4. Unreported analytic uncertainty and repeatability. | 1. Resample or re-bin spectra to common wavenumber grid; convert units on ingest.  2. Descriptor extraction. Compute a shared set of engineered descriptors (e.g. carbonyl index, integrated band areas, peak ratios, etc.) to act as semantic anchors.  3. Batch correction and calibration transfer: ComBat, quantile mapping, or simple linear shift and scale per site; use reference standards for calibration.  4. Uncertainty. Record per-sample analytical CV and propagate via heteroscedastic and Bayesian models. |
| Image/ morphology data | 1. Varying pixel scale or magnification or field of view result in scale mismatch.  2. Contrast, brightness, and detector settings differ; charging or artefacts and compression losses.  3. Inconsistent preprocessing and segmentation parameters; inconsistent area and roughness metrics.  4. Missing or undocumented acquisition parameters. | 1. Record acquisition metadata for every image, such as pixel size, mag, acceleration voltage, detector type.  2. Resample and normalize spatial scale. Resample images to a common pixel or compute scale-normalized descriptors.  3. Use reproducible segmentation pipelines. Otsu or adaptive threshold with fixed SE size; publish code or containerized pipeline.  4. Scale-invariant features and augmentation. SIFT-like descriptors, texture metrics, synthetic augmentation for domain coverage; quantify reproducibility via reference images and CV. |

**Table S2. Representative descriptors and cross-modal semantic alignment mapping for MP aging.**

| **Underlying Physicochemical Phenomenon** | **Modalities involved** | **Representative Descriptors** | **Cross-Modal Alignment Target** | **Embedded Physical and Kinetic Constraints** |
| --- | --- | --- | --- | --- |
| Surface photo-oxidation | FT-IR, Raman | Carbonyl Index (CI), Hydroxyl (OH) Index, crack density | Latent Oxidation Degree: Aligns spectroscopic functional group intensities with elemental composition. | Pseudo-first-order accumulation kinetics;  Arhenius-type temperature dependence of rate constant |
|  | XPS / EDS elemental analysis | Surface O/C atomic ratio, relative abundance of C–O and C=O bonding states |  |  |
| Mechanical embrittlement and fragmentation | SEM, AFM | Surface roughness (R_a_, R_q_), micro-crack density, fractal dimension of particle edges | Latent Fragmentation Propensity: Aligns morphological degradation features with macroscopic volumetric size shifts. | Weibull distribution logic for particle size reduction over time;  Stress-strain degradation models; |
|  | Laser diffraction, DLS | D_10_/D_50_/D_90_, projected area (µm²), aspect ratio |  |  |
| Hydrophobicity alteration and additive leaching | Contact Angle Goniometry | Water Contact Angle (WCA, degrees), surface free energy | Latent Interfacial Affinity: Aligns bulk hydrophilicity changes with the precise quantification of leached small molecules. | Diffusion-controlled additive release models based on Fick’s law;  Consistency between predicted interfacial affinity and additive diffusion flux |
|  | GC-MS, HPLC | Concentration of leached additives, additive depletion rates |  |  |
| Bioavailability and corona | CLSM, Fluorescence Microscopy | Biofilm thickness, surface coverage, live/dead cell ratio, | Latent Corona Maturation and Shielding Effect: Aligns physical biofilm morphology with biochemical EPS composition and interfacial charge. | Monod kinetics for biofilm growth, and saturation limits for EPS deposition.;  Regularization linking biochemical composition to corona growth dynamics;  Constraint coupling charge variation with biofilm deposition dynamics |
|  | LC-MS, multi-omics profiling | EPS protein, carbohydrate ratios, metabolite fingerprints, microbial functional markers |  |  |
|  | Zeta potential, QCM-D | Surface charge shift, deposition rate of natural organic matter |  |  |

**Table S3 Physicochemical laws relevant to MP aging and their integration into modeling frameworks.**

| **Physicochemical Law and Principle** | **Equation** | **Associated MP Aging Process** | **Integration into Modeling Framework** |
| --- | --- | --- | --- |
| Mass Conservation | ∂C/∂t + ∇·J = S | Governs all transport, transformation, and mass exchange processes. | Enforce as hard constraint or penalty to prevent non-physical mass creation and loss. |
| Fick’s First Law | J = -D ∇C | Diffusive transport of additives and dissolved species. | Used to parameterize flux and diffusion coefficient D constrained via priors or fitted within physics-informed loss. |
| Fick’s Second Law | ∂C/∂t = D ∇²C + R | Time-dependent release and transport processes. | Embed as PDE constraint or regularize predicted concentration dynamics. |
| Stokes’ Law | $V_{s}=\frac{2\left( \rho_{P}-\rho_{f} \right)gr^{2}}{9\mu}$ | Particle sedimentation and vertical transport. | Constrain particle transport velocity as function of size and density. |
| Advection–Dispersion Equation (ADE) | ∂C/∂t + u ∂C/∂x = D ∂²C/∂x² + S | Transport in aquatic and porous systems. | Incorporate as governing transport equation and learn parameters under physical constraints. |
| Arrhenius Equation | k(T) = A exp(-Ea / (R T)) | Temperature dependence of reaction and degradation rates. | Constrain rate constants and treat A and E_a_ as learnable but physically bounded parameters. |
| First-Order / Saturation Kinetics | dX/dt = -k X  $X=X_{\infty}(1-e^{-kD})$ | Chemical aging. | Embed as functional form for descriptor evolution and add physics penalty. |
| Weibull Distribution | S(t) = exp[-(t/λ) ^k^] | Mechanical degradation and particle breakage. | Model fragmentation probability and couple PSD evolution to Weibull parameters. |
| Paris Law | da/dN = C (ΔK)^m^ | Crack propagation under cyclic stress. | Constrain crack growth rate under repeated mechanical stress. |
| Archard Wear Law | $V=\frac{KLS}{H}$ | Surface wear and abrasion-induced fragmentation. | Link mechanical stress to material loss and use as prior in fragmentation models. |
| Hooke’s Law | σ = Eε | Stress–strain behavior leading to embrittlement. | Define thresholds for elastic-to-fracture transitions. |
| Smoluchowski Coagulation Equation | $\frac{{dn}_{k}}{dt}=\frac{1}{2}\sum_{i+j=k} K_{ij}n_{i}n_{j}-n_{k}\sum_{j} K_{kj}n_{j}$ | Particle aggregation and size distribution evolution. | Incorporated to constrain particle size distribution (PSD) dynamics. |
| DLVO Theory | V_tot_ = V_vdW_ + V_el_ | Colloidal stability, aggregation and dispersion. | Constrain aggregation probability based on surface interactions and zeta potential. |
| Henry’s Law | C_g_ = HC_l_ | Gas-liquid partitioning of volatile compounds. | Constrain phase transfer between gas and aqueous phases. |
| Langmuir Isotherm | $q=\frac{q_{max}KC}{1+KC}$ | Adsorption of contaminants or NOM onto MP surfaces. | Constrain surface loading and saturation behavior. |
| Freundlich Isotherm | q = K_F_C^1/n^ | Nonlinear adsorption behavior. | Flexible empirical constraint for heterogeneous adsorption. |
| Langmuir–Hinshelwood Kinetics | Combined adsorption–reaction model | Surface-mediated photochemical reactions. | Couple adsorption equilibrium with reaction kinetics in surface reaction modules. |
| Beer–Lambert Law | I = I_0_e^-αx^ | Light attenuation affecting photodegradation. | Correct effective UV exposure in kinetic models. |
| Higuchi Model | M_t_ / M_∞_ ∝ t^1/2^ | Diffusion-controlled release from polymers. | Constraint for early-stage additive leaching. |
| Korsmeyer–Peppas Model | M_t_ / M_∞_ = kt^n^ | Generalized release kinetics. | Infer release mechanisms via exponent *n* and incorporate as parametric template. |
| Stokes–Einstein Equation | $D=\frac{K_{B}T}{6 \pi\eta r}$ | Molecular diffusion in fluids. | Provide prior estimates for diffusion coefficients. |
| Nernst–Planck Equation | $J_{i}={-D}_{i}{\nabla C}_{i}-\frac{z_{i}D_{i}F}{RT}C_{i}\nabla\phi$ | Ion transport with electromigration. | Couple diffusion and electromigration in charged environments. |
| Darcy’s Law | q = -(K / μ) ∇ P | Flow through porous media. | Constrain fluid-driven transport in soils and sediments. |
| Deposition–Filtration Models | ∂C/∂t = - λ_d_C | Particle retention and deposition in porous media. | Model particle removal and retention rates. |
| Monod Kinetics | μ = μ_max_ S / (K_s_ + S) | Microbial growth and biodegradation. | Constrain biologically mediated degradation rates. |
| Michaelis–Menten Kinetics | v = V_max_ [S] / (K_M_ + [S]) | Enzymatic degradation processes. | Apply to enzyme-driven polymer breakdown. |
| Power-Law Scaling (PSD) | n(r) ∝ r^-α^ | Particle size distributions in fragmentation systems. | Use as prior for PSD shape. |
| Buoyancy (Archimedes Principle) | F_b_ = ρ_f_ V_g_ | Floating vs sinking behavior. | Determine transport regime boundaries. |
| Quantum Yield / Photochemical Efficiency | Rate ∝ Φ · I_abs_ | Photochemical degradation efficiency. | Link absorbed photons to reaction rates. |
| Reynolds Number / Shear Stress Scaling | $Re=\frac{pvL}{\mu}, \tau\sim\rho u_{*}^{2}$ | Hydrodynamic stress driving abrasion. | Trigger or scale mechanical degradation processes. |
